# Biomimicking Fiber Scaffold as an Effective In Vitro and In Vivo MicroRNA Screening Platform for Directing Tissue Regeneration

**DOI:** 10.1002/advs.201800808

**Published:** 2019-02-27

**Authors:** Na Zhang, Ulla Milbreta, Jiah Shin Chin, Coline Pinese, Junquan Lin, Hitomi Shirahama, Wei Jiang, Hang Liu, Ruifa Mi, Ahmet Hoke, Wutian Wu, Sing Yian Chew

**Affiliations:** ^1^ School of Chemical and Biomedical Engineering Nanyang Technological University 62 Nanyang Drive Singapore 637459 Singapore; ^2^ NTU Institute of Health Technology Interdisciplinary Graduate School Nanyang Technological University Singapore 639798 Singapore; ^3^ Artificial Biopolymers Department Max Mousseron Institute of Biomolecules (IBMM) UMR CNRS 5247 University of Montpellier Faculty of Pharmacy Montpellier 34093 France; ^4^ School of Life Sciences and Medical Center University of Science and Technology of China Hefei Anhui 230027 P. R. China; ^5^ Department of Neurology Johns Hopkins University School of Medicine Baltimore MD 1521205 USA; ^6^ Guangdong‐Hongkong‐Macau Institute of CNS Regeneration Ministry of Education CNS Regeneration Collaborative Joint Laboratory Jinan University Guangzhou 510632 P. R. China; ^7^ Re‐Stem Biotechnology Co., Ltd. Suzhou 330520 P. R. China; ^8^ Lee Kong Chian School of Medicine Nanyang Technological University Singapore 308232 Singapore

**Keywords:** contact guidance, electrospinning, gene silencing, neural tissue engineering, RNA interference

## Abstract

MicroRNAs effectively modulate protein expression and cellular response. Unfortunately, the lack of robust nonviral delivery platforms has limited the therapeutic application of microRNAs. Additionally, there is a shortage of drug‐screening platforms that are directly translatable from in vitro to in vivo. Here, a fiber substrate that provides nonviral delivery of microRNAs for in vitro and in vivo microRNA screening is introduced. As a proof of concept, difficult‐to‐transfect primary neurons are targeted and the efficacy of this system is evaluated in a rat spinal cord injury model. With this platform, enhanced gene‐silencing is achieved in neurons as compared to conventional bolus delivery (*p* < 0.05). Thereafter, four well‐recognized microRNAs (miR‐21, miR‐222, miR‐132, and miR‐431) and their cocktails are screened systematically. Regardless of age and origin of the neurons, similar trends are observed. Next, this fiber substrate is translated into a 3D system for direct in vivo microRNA screening. Robust nerve ingrowth is observed as early as two weeks after scaffold implantation. Nerve regeneration in response to the microRNA cocktails is similar to in vitro experiments. Altogether, the potential of the fiber platform is demonstrated in providing effective microRNA screening and direct translation into in vivo applications.

## 1 Introduction

MicroRNAs (miRs) are short noncoding RNAs that effectively regulate protein translation by enabling mRNA degradation and/or repressing protein translation.^1^, ^2^ This process plays significant roles in modulating protein expression by RNA interference (RNAi). Correspondingly, miRs have been recognized as powerful therapeutic agents with high efficacy in modulating cell fate and numerous efforts have been placed on screening differential expressions of miRs in disease contexts in hope of identifying promising therapeutics.^3^, ^4^

While the potential of miRs as promising therapeutics for tissue regeneration (cartilage,^5^ skeletal muscle,^6^ and bone^7^) is well known and numerous libraries of promising miR candidates have been created,^8^, ^9^ several bottlenecks remain and have been preventing the successful utilization of miR therapy.^10^ First and foremost, is the lack of robust nonviral platforms to deliver miRs effectively in vitro and in vivo.^11^, ^12^ Although nanoparticles have been utilized to deliver miRs,^13^, ^14^ repeated administrations were almost always required to achieve long‐term gene silencing in vivo.^15^ Given this limitation, biodegradable hydrogel is increasingly employed as a platform for sustained delivery of miRs to facilitate tissue regeneration.^16^, ^17^, ^18^ However, hydrogels are often isotropic in architecture and hence lack the ability to direct tissue regrowth.^19^ Second and equally important, is the fact that despite the plethora of in vitro drug screening platforms available, such as micropillars^20^ and microfluidic devices,^21^ it remains difficult to translate in vitro outcomes to in vivo applications with good correlations.^22^, ^23^, ^24^, ^25^ Consequently, the development of effective miR therapeutics, particularly targeting tissue regeneration, remains slow.

Here, we introduce a biomimicking aligned fiber platform which allows the delivery of miRs both in vitro and in vivo in a sustained and nonviral manner. The easy incorporation of different miRs into this platform also makes it possible to conduct extensive miR screening. Furthermore, this fiber‐miR delivery system can be easily translated into a 3D configuration by incorporating it with collagen hydrogel, which further extends its application for in vivo miR screening and directing tissue regeneration. To evaluate the robustness of this platform, we used a stringent criterion by targeting difficult‐to‐transfect primary neurons of different ages from both the central and peripheral nervous systems (CNS and PNS, respectively). We further evaluated the in vivo performance of the fiber system by using a complete transection spinal cord injury (SCI) model as a proof of concept.

In particular, we modulated the intrinsic growth ability of neurons by targeting axon local protein synthesis. Within the body, axons can extend for long distances of up to 3 m. For efficient cell function, protein synthesis occurs locally at the terminal and growth ends of axons so that the transport of biomolecules from the main cell body (i.e., cell soma) is not critical. Such controlled local protein synthesis at the ends of axons (a.k.a. distal axons)^26^ has allowed severed axons to undergo regeneration within hours after injuries.^26^, ^27^ This local protein synthesis is finely controlled by miRs, and miR‐21,^28^ miR‐222,^29^ miR‐132,^30^ and miR‐431^31^ have been identified to enhance nerve regeneration when they are overexpressed in cultured neurons independently.^28^, ^29^, ^32^ Specifically, MiR‐431 enhances Wnt signaling, which is required for neurogenesis and axon growth by decreasing the expression of a Wnt antagonist, Kremen1.^31^ In addition, miR‐431 could regulate motor neuron neurite length by targeting chondrolectin, one of several molecules that plays a role in motor neuron axon outgrowth.^33^ MiR‐21, on the other hand, targets Sprouty2, which in turn inhibits the Ras/Raf/extracellular signal‐regulated kinase (ERK) pathway, and antagonizes fibroblast growth factor (FGF) signaling.^28^ Mir‐222 targets phosphatase and tensin homolog (PTEN), an inhibitor of the PI3K pathway that is important to central axon growth.^29^ Finally, Ras GTPase, p120RasGAP (Rasa1), which is involved in cytoskeletal regulation, is targeted by miR‐132.^34^

Therefore, in contrast to the conventional ways of simply modulating the microenvironment of injured neurons with neurotrophic factors^35^ or neutralizing growth inhibitory molecules,^36^ we recognize that mature neurons possess diminished regenerative capacity, which is a major cause of regeneration failure. Hence, the mere modulation of the cell/tissue microenvironment may not be sufficient to achieve the desired regeneration outcomes.^27^

Specifically, we hypothesized that the incorporation of miRs into biomimicking polycaprolactone (PCL) fiber constructs would enhance gene silencing by localized and prolonged availability of miRs. Furthermore, by taking advantage of the biomimicry nature of fibers, physiologically relevant behaviors could be elicited in cells in vitro; hence allowing good correlation between in vitro and in vivo biological behaviors. We show that such fiber‐mediated miR delivery platform may serve as effective in vitro and in vivo drug testing systems.

## 2 Results

### 2.1 Characterization, Cellular Response, and Cy5‐RNA Distribution on PCL Aligned Fibers

**Figure 1:**
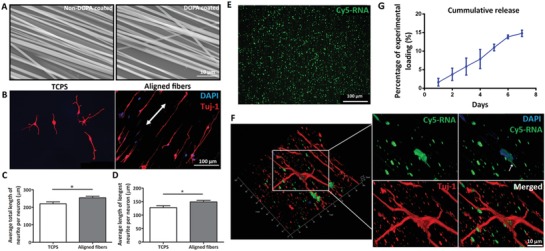
Characterization of aligned fiber substrates. A) Uncoated fibers (fiber diameter φ = 790 ± 152 nm) and poly‐DOPA coated fibers (φ = 948 ± 127 nm). B) E14 cortical neurons cultured for 3 d on aligned fibers versus tissue culture plates (TCPS). C,D) Average total length of neurite and average length of the longest neurite, showing that the aligned topography significantly promoted neurite extensions. *p < 0.05, Student's *t*‐test. E) Fluorescent microscopy image shows a uniform distribution of Cy5‐RNA on aligned fiber substrates. F) Cy5 signals were clearly detected in the cultured P1 cortical neurons, indicating successful cellular uptake. G) Release profile of microRNAs from the aligned fiber substrates.

The PCL fibers were produced using an electrospinning technique. **Figure** **1**A shows the morphology of the aligned fibers before and after poly‐3,4‐dihydroxy‐l‐phenylalanine (DOPA) coating. Poly‐DOPA coated fibers had an average diameter of 948 ± 127 nm while uncoated fibers had an average fiber diameter of 790 ± 152 nm. This suggests that poly‐DOPA coating did not alter the topography of these aligned fibers (*p* > 0.05).

To evaluate the effect of aligned fiber topography on neurite outgrowth, E14 cortical neurons were cultured on tissue culture plates (TCPS) and aligned fibers for comparison. Three days after culture, aligned neurite extensions were detected on the fiber substrates. Conversely, cells that were cultured on TCPS grew toward different directions (Figure 1B). As indicated in Figure 1C,D, cortical neurons cultured on aligned fibers exhibited significantly longer neurite outgrowth in terms of the total length and the longest length of neurites as compared to conventional 2D cultures.

A uniform distribution of Cy5‐labelled double‐stranded RNA (Cy5‐RNA), which was of similar size as miRs, was clearly identified on the aligned fiber substrates after 2 h of coating (Figure 1E). Three days after cell seeding onto these Cy5‐RNA absorbed scaffolds, Cy5‐RNA signals were detected in the neurons, suggesting that the miRs were taken up successfully by the cortical neurons (Figure 1F).

The average loading efficiency of miRs on the aligned fiber scaffolds was around 77%. Specifically, 15% of miRs were released at Day 7. As can be seen from the release curve, a sustained delivery of miRs was achieved by our aligned fiber platform (Figure 1G).

### 2.2 Systematic In Vitro Screening for Optimal miR Combinations to Promote Neurite Outgrowth

**Figure 2:**
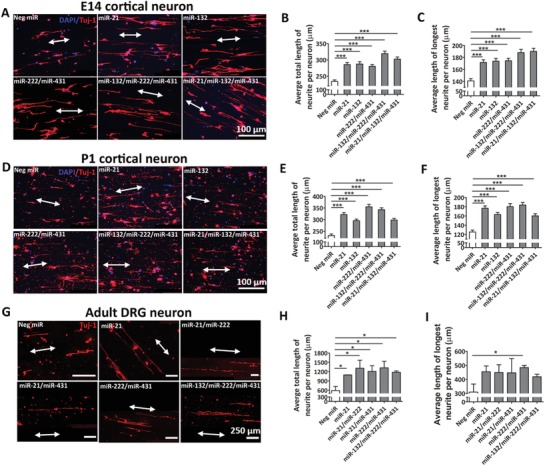
In vitro miR cocktail screening using E14, P1 cortical neurons and adult DRG neurons. A) Representative fluorescent microscopy images of E14 cortical neurons. B,C) Average total neurite length and average length of longest neurite of the top five groups of E14 cortical neurons. D) Representative fluorescent microscopy images of P1 cortical neurons. E,F) Average total neurite length and average length of longest neurite of the top five groups of P1 cortical neurons. G) Representative fluorescent microscopy images of adult DRG neurons. H,I) Average total neurite length and average length of longest neurite of the top five groups of adult DRG neurons. *p < 0.05, **p < 0.01, ***p < 0.001, One‐way ANOVA/Kruskal–Wallis test and Mann–Whitney post hoc test.

Here, we analyzed all possible combinations of the four miRs to identify the optimal cocktails that could maximize neurite outgrowth. In order to ensure the robustness of the miR mixtures in enhancing regeneration from neurons, we utilized neurons of different ages (embryonic day 14 (E14) vs postnatal day 1 (P1) vs adult) and origin (CNS vs PNS). **Figure** **2** summarizes the top five cocktails that were identified using the aligned fiber screening platforms. In all samples, neurite projections were found in both directions, parallel to the aligned fibers (Figure 2A,D,G, white arrows). Such aligned neurites enabled easy tracing and quantification of neurite outgrowth lengths.

#### 2.2.1 Embryonic Cortical Neurons

As shown in Figure 2A–C, scrambled Neg miR (Neg miR) induced a baseline total length of 233.7 ± 4.711 µm and a longest length of 140.8 ± 3.744 µm. These measurements were not significantly different as compared to untreated cells and cells that were treated with transfection reagent, TransIT‐TKO (TKO) (Figure S1A–C, Supporting Information).

As shown in Figure S1A,D,E in the Supporting Information, for treatment with individual miRs, neurite outgrowth was similar for miR‐21 (285.3 ± 6.721 µm) and miR‐132 (287.2 ± 7.064 µm). These miR treatments, in turn, resulted in longer neurite outgrowths than miR‐222 (233.5 ± 5.352 µm) and miR‐431 (246.6 ± 5.352 µm).

For treatment with two‐miR cocktails, miR‐222/miR‐431 showed the best result followed by miR‐132/miR‐431 and miR‐21/miR‐132. Among these six groups, miR‐21/miR‐431 resulted in the smallest extent of neurite outgrowth (Figure S1A,F,G, Supporting Information).

As depicted in Figure S1A,H,I in the Supporting Information, for three‐miR cocktails, the withdrawal of miR‐21 resulted in the longest neurite extension followed by the withdrawal of miR‐222. Finally, the combination of four miRs did not generate the best outcome (Figure S1J,K, Supporting Information).

Taken together, the top five groups that induced the longest neurite outgrowth in E14 cortical neurons were: miR‐132/miR‐222/miR‐431 > miR‐21/miR‐132/miR‐431 > miR‐132 > miR‐21 > miR‐222/miR‐431 (Figure 2A–C).

#### 2.2.2 P1 Cortical Neurons

As shown in Figure 2D–F, similar to the embryonic cortical neurons, Neg miR treatment induced a baseline in terms of total length (231.3 ± 6.577 µm) and the longest length of neurites (125.4 ± 3.997 µm). These values obtained were similar to the embryonic cortical neurons and did not show significant difference as compared to untreated cells (249.6 ± 6.188 µm) and cells treated with TKO only (228.2 ± 6.776 µm) (Figure S2A–C, Supporting Information).

For treatment with individual miRs (Figure S2A,D,E, Supporting Information), similar trends were observed as compared to embryonic cortical neurons. Specially, miR‐21 (321.3 ± 8.264 µm) resulted in the longest neurite outgrowth, followed by miR‐132 (295.3.2 ± 6.348 µm). MiR‐222 (251.7 ± 5.411 µm) and miR‐431 (269.1 ± 7.135 µm) were similar and more inferior.

As shown in Figure S2A,F,G in the Supporting Information, for two‐miR cocktails, miR‐222/miR‐431 showed the best result followed by miR‐132/miR‐431 and miR‐21/miR‐132. Among these six groups, miR‐21/miR‐431 was the worst. These trends were consistent with those observed in embryonic cortical neurons.

For three‐miR cocktails, the withdrawal of miR‐21 exhibited the longest neurite extension followed by the withdrawal of miR‐222 (Figure S2A,H,I, Supporting Information). Finally, the combination of four miRs did not generate the best outcome (Figure S2J,K, Supporting Information).

Altogether, the top five groups that induced the longest neurite outgrowth in P1 cortical neurons were: miR‐222/miR‐431 > miR‐132/miR‐222/miR‐431 > miR21 > miR132 > miR‐21/miR‐132/miR‐431 (Figure 2D–F). These groups were similar to those obtained when embryonic cortical neurons were evaluated, although the ranking of the extent of neurite outgrowth is slightly different.

#### 2.2.3 Adult Dorsal Root Ganglion (DRG) Neurons

As shown in Figure 2G–I, Neg miR induced a baseline total length of 591.0 ± 132.7 µm and a longest length of 309.2 ± 57.4 µm. Similar to cortical neurons, these measurements were not significantly different as compared to untreated cells and cells that were treated with TKO only (Figure S3A–C, Supporting Information).

As shown in Figure S3A,D,E in the Supporting Information, for treatment with individual miRs, miR‐21 (1091.9 ± 6.0 µm) resulted in longer neurite outgrowths again, an observation that was similarly made with the cortical neurons. However, in contrast to cortical neurons, miR‐431 (1080.8 ± 161.7 µm) resulted in similar and longer neurite outgrowths as miR‐21, followed by miR‐132 (1057.5 ± 161.8 µm). The worst was observed with miR‐222 treatment (956.6 ± 97.0 µm).

For treatment with two‐miR cocktails, only slight similarity to cortical neurons was observed. Specially, miR‐222/miR‐431 showed the best result. However, this was then followed by miR‐21/miR‐222 and miR‐21/miR‐431, which was different from cortical neurons. Among these six groups, miR‐132/miR‐431 resulted in the smallest extent of neurite outgrowth (Figure S3A,F,G, Supporting Information).

As demonstrated in Figure S3A,H,I in the Supporting Information, for three‐miR cocktails, the withdrawal of miR‐21 resulted in the longest neurite extension again. This was then followed by the withdrawal of miR‐132. Finally, consistent with the results from cortical neurons, the combination of four miRs did not generate the best outcome (Figure S3J,K, Supporting Information).

**Figure 3:**
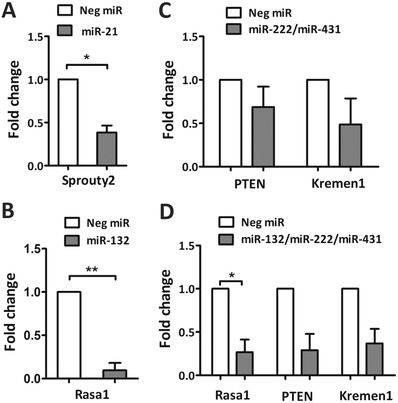
Target gene knockdown in P1 cortical neurons cultured on aligned fiber substrates at day 3 in vitro. A) Fold change of Sprouty2 after treatment with miR‐21. B) Fold change of PTEN after treatment with miR‐132. C) Both PTEN (*p* = 0.3145) and Kremen1 (*p* = 0.2287) were knocked down after treatment with miR‐222/miR‐431. D) Rasa1, PTEN (*p* = 0.0635) and Kremen1 (*p* = 0.0645), were knocked down following miR‐132/miR‐222/miR‐431 treatment. All comparisons were normalized with Neg miR‐treated group. *p < 0.05, **p < 0.01, Student's *t*‐test.

Taken together, the top five groups that induced the longest neurite outgrowth in adult DRG neurons were: miR‐222/miR‐431 > miR‐21/miR‐222 > miR‐21/miR‐431 > miR‐132/miR‐222/miR‐431 > miR‐21 (**Figure** **3**G–I). Correspondingly, miR‐21, miR‐222/miR‐431, and miR‐132/miR‐222/miR‐431 appeared to enhance neurite outgrowth in neurons, regardless of the age and origin of neurons.

### 2.3 Aligned Fiber‐Mediated miR Delivery Induced Target Gene Silencing

The target gene silencing effect was evaluated 3 d after scaffold‐mediated miR transfection in P1 cortical neurons. Considering cell viability and in vivo relevance, P1 cortical neurons were chosen. Based on the screening data of P1 cortical neuron, the top four groups (miR‐21, miR‐132, miR‐222/miR‐431, and miR‐132/miR‐222/miR‐431) were selected and the gene expression levels of their downstream targets were determined.

As compared to Neg miR treatment, the transfection of miR‐21 and miR‐132 resulted in significant knockdown of their downstream targets, Sprouty2 (Figure 3A) and Rasa1 (Figure 3B), respectively. The gene silencing effect of miR‐222/miR‐431, however, was not as obvious as the treatment with single miRs (Figure 3C). On the other hand, the knockdown effect in miR‐132/miR‐222/miR‐431 treated group was more significant, especially for Rasa1 (Figure 3D).

As compared to 2D bolus delivery (Figure S4, Supporting Information), the miRs in each group exhibited significantly better gene silencing effects when cells were transfected using the fiber scaffolds.

### 2.4 MiR Treatment Enhanced the Formation of Growth Cones

**Figure 4:**
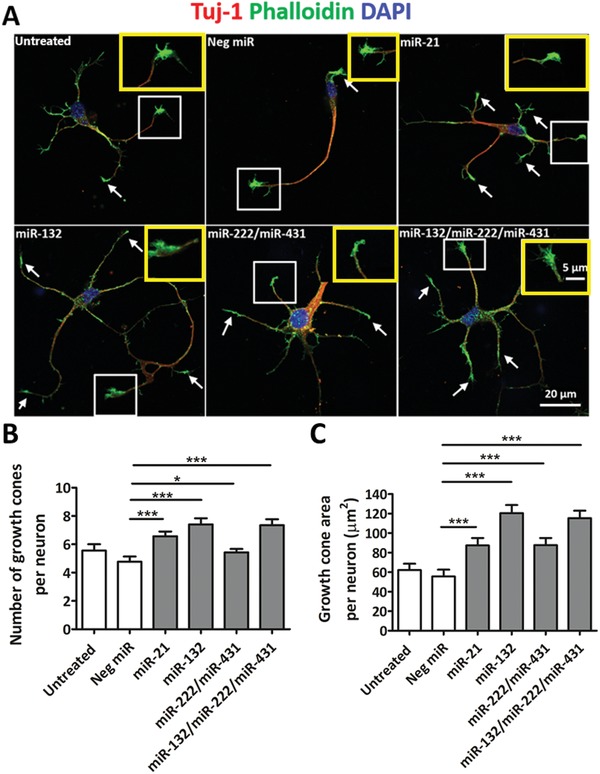
Growth cone quantification following miR treatment. A) Representative confocal images of immunofluorescent‐stained neurons. White arrows represent growth cones. Yellow boxes depict high magnification images of growth cones within white boxes. B) Number of growth cones and C) area of growth cone per neuron, suggested that the treatment of miR/miR cocktails significantly promoted growth cone formation. *p < 0.05, **p < 0.01, ***p < 0.001, Shapiro–Wilk normality test followed by Kruskal–Wallis test and Mann–Whitney post hoc test.

Besides evaluating target gene knockdown in the top four groups of miRs, we also analyzed the effects of these miRs on growth cone formation by immunofluorescent staining on 2D cultures so as to gain insight to the possible mechanisms that may be involved in this miR‐related enhanced neurite outgrowth. As shown in **Figure** **4**A, three days after cell seeding, the growth cone structures were located at the tips of the neurites (pointed by the arrows). The number of growth cones and the growth cone area were quantified. Correspondingly, miR‐132 and miR‐132/miR‐222/miR‐431‐treated neurons exhibited more growth cones as compared to Neg miR‐treated neurons, followed by the miR‐21‐treated group (Figure 6B). Statistical analysis of the growth cone area showed that miR‐132‐and miR‐132/miR‐222/miR‐431‐treated neurons also exhibited larger growth cone areas as compared to Neg miR treatment. This was then followed by miR‐21‐and miR‐222/miR‐431‐treated groups (Figure 4C).

### 2.5 In Vivo Scaffold Characterization

**Figure 5:**
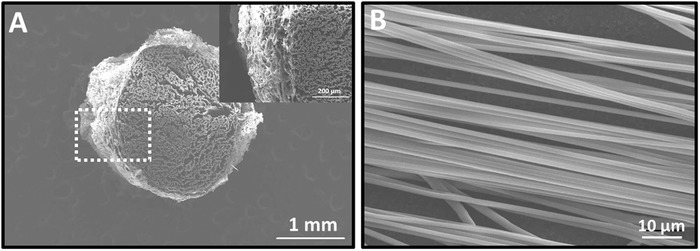
In vivo scaffold design. A) SEM image of cross‐section of fiber‐hydrogel scaffold, showing that aligned fibers were uniformly distributed and well‐oriented inside the collagen matrix. B) SEM image of aligned fibers with an average diameter of 1.245 ± 0.13 µm.

To translate the axon‐growth promoting aligned fibers to implantable constructs, we utilized collagen to support aligned fibers to formulate a 3D structure. **Figure** **5**A shows the cross‐section view of fiber‐hydrogel scaffold. This scaffold comprised of electrospun aligned fibers that were supported by a collagen matrix. The presence of collagen maintained the fiber orientation and alignment without inducing fiber fusion. The aligned fibers possessed an average diameter of 1.245 ± 0.13 µm, as shown in Figure 5B.

### 2.6 MiR Treatment Improved Nerve Ingrowth into the Fiber‐Hydrogel Scaffold after SCI

**Figure 6:**
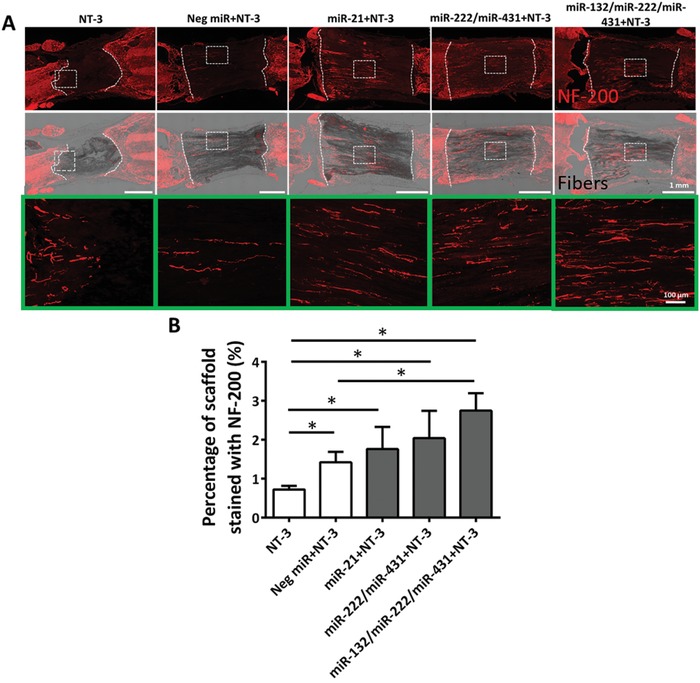
MiRs stimulated extensive neurite ingrowth into scaffolds 2 weeks after SCI. A) Representative fluorescent microscopy images of NF‐200 (red) expression at injury sites in the various groups. White dotted boxes represent the region of enlargement (green boxes). B) Quantification of the percentage of scaffold stained with NF‐200 suggested that the presence of miRs significantly promoted nerve regeneration after SCI. *p < 0.05, Shapiro–Wilk normality test followed by Kruskal–Wallis test and Mann–Whitney post hoc test.

To evaluate the in vivo efficacy of our 3D aligned fiber scaffold, we used a complete transection SCI model as a proof of concept. Based on our in vitro screening data from Section 2.2, we chose the top single, double and triple miR combinations for in vivo study. These miR combinations were coupled with Neurotrophin‐3 (NT‐3) as previous SCI studies have suggested that the presence of NT‐3 is crucial for promoting neuronal survival, axonal sprouting, and regeneration.^37^, ^38^, ^39^, ^40^ However, we found that NT‐3 alone was insufficient to stimulate robust neurite ingrowth into the scaffold after complete spinal cord transection (**Figure** **6**).

On the other hand, when NT‐3 was coupled with the selected miR combinations (Figure 6), extensive neurofilament ingrowth into the scaffolds was observed, especially for the triple miR treatment. Specifically, neurofilaments were evenly distributed inside the scaffolds. Furthermore, the aligned fibers provided topographical guidance to direct regenerated neurofilaments in the direction that was parallel to the spinal cord. Similar to in vitro observations, there was very limited neurofilament ingrowth into the scaffolds that incorporated Neg miR (Figure 6A,B).

### 2.7 MiR Treatment Did Not Induce Obvious Glial Scar Formation

To evaluate any possible effects of the miR cocktails on glial scarring, we analyzed glial scar formation by glial fibrillary acidic protein positive (GFAP^+^) signals. As shown in Figure S5 in the Supporting Information, the implantation of fiber‐hydrogel scaffolds showed no difference in glial scarring regardless of miR combinations.

## 3 Discussion

Neural tissue engineering approaches, such as using nerve grafts and biomaterial scaffolds, or using cells and neurotrophic factors have emerged as alternatives for nerve injury treatment.^41^ Evidences have indicated that the combination of these therapies could better mimic the properties of the microenvironment that is required for nerve repair.^42^

Electrospun fibers have been shown to mimic the architecture of the natural extracellular matrix and provide the necessary topographical cues to modulate cell fate.^43^, ^44^, ^45^ Specifically, electrospun fibers could mimic the size scale and architecture of axons, which allows controlled and sustained delivery of biomolecules to direct oligodendrocyte progenitor cell (OPC) differentiation, maturation^43^ and myelination.^46^

As small noncoding RNAs that effectively modulate cellular behaviors, miRs have emerged as one of the most prominent biomolecules with powerful therapeutic potential. Correspondingly, numerous screening studies have established libraries of dysregulated miRs in the context of diseases and injuries in hope to develop effective treatments.^9^, ^47^, ^48^ Unfortunately, the use of miRs for therapeutic purposes remain limited. We speculate that this is partly due to the lack of robust nonviral platforms to deliver miRs efficiently in vitro and in vivo; and the shortage of effective drug screening devices that correlate in vitro outcomes with in vivo performances. With these requirements in mind, we developed a robust biomimicking fiber platform, which could deliver miRs in a sustained (Figure 1G) and nonviral manner using PCL electrospun fibers. PCL was utilized due to its biocompatibility and is also a U.S. Food and Drug Administration (FDA) approved polymer. As such, PCL has been widely used for both in vitro cell culture^49^ and in vivo implantation.^50^ By applying this screening platform, we showed that even sensitive and notoriously difficult to transfect cells, like neurons, could be modulated. In addition, the efficacy of this platform was further evaluated in a rat spinal cord transection model as a proof of concept. The biomimicry nature of the system provided good biological correlation between in vitro and in vivo outcomes.

As compared to conventional 2D cultures, our aligned fiber platform provided contact guidance to orientate and promote the growth of neurites. Consequently, significantly longer neurite outgrowth lengths were achieved in E14 cortical neurons (Figure 1B–D). Notably, this is the first time that CNS neurons were tested on aligned fibers versus 2D cultures. Regardless, this result is consistent with the observations that have been reported by other groups when DRG neurons from the PNS were cultured on aligned fibers versus 2D film controls.^51^, ^52^ Furthermore, the configuration of our aligned fiber platform is convenient for imaging and quantification due to the obvious growth direction of the neurites.

By coating the fibers with poly‐DOPA, various biomolecules such as laminin and miRs, which facilitate neuron attachment and neurite extension respectively, can be adsorbed onto the scaffolds. As a mussel‐inspired bioadhesive,^53^ poly‐DOPA has been used for sustained delivery of miRs^43^, ^54^ and siRNAs.^55^ Poly‐DOPA coated substrates allowed the efficacious absorption of the miRs onto the aligned fibers without altering fiber topography (Figure 1A) or the bioactivity of the miRs. Moreover, the sustained delivery of miRs from the aligned fibers reduced cytotoxicity^43^ and facilitated better gene silencing outcomes as compared to conventional bolus delivery (Figure 3 vs Figure S4, Supporting Information). To identify the amount of miRs that is required to exert effective downstream gene silencing in primary neurons, we carried out a titration process and compared 0.25, 0.5, and 1 µg of miRs. From these preliminary studies, 1 µg of miRs was found to be an effective amount for gene regulation in primary neurons and was hence utilized for our actual experiments despite that the loading efficiency on the fibers was around 77%.

Besides establishing a novel miR‐screening substrate that correlates well in vitro and in vivo, we also introduced a new approach to enhance nerve regeneration after SCI. SCI lead to devastating outcomes of paralysis and functional impairment and are the major causes of morbidity and mortality, particularly in young adults and children.^56^ Specifically, instead of the conventional methods of modulating the microenvironment after injury with overexpression of growth factors^35^ and neutralizing any growth inhibitory molecules,^36^ we targeted the intrinsic growth ability of neurons by enhancing local protein synthesis at the growth cones of axons using miRs. Here, the miRs of interest, miR‐21, miR‐222, miR‐132, and miR‐431, have been shown to increase significantly at the growth cones of neurons after nerve injuries.^28^, ^29^, ^30^, ^31^ The upregulation in expression of these miRs modulates different pathways in neurons, resulting in robust axon regeneration.^29^, ^57^ In particular, they either enhance pathways that regulate neurogenesis and axon growth or directly remove molecular brakes that prevent regrowth.^28^, ^29^, ^31^, ^33^, ^34^

Based on our previous work with OPCs where miR‐219/miR‐338 cocktail transfection enhanced differentiation outcomes more than miR‐219 treatment alone,^43^ we speculated that a combination of miRs may also benefit axon regeneration. Therefore, to further understand the capabilities of these miRs and their effects on neurons, we proceeded to investigate the possible synergistic or antagonistic effects of these miRs by using our aligned fiber miR screening platform.

Our miR screening data showed that there are many similar miR combinations for all three types of neurons, regardless of their age and origin. Adult DRG neurons exhibited more robust neurite outgrowth and greater enhancement with miR treatment than CNS neurons. Interestingly, the enhancement in neurite length of DRG neurons after miR treatment, as observed in this work, was stronger (1.8–2.2 times) as compared to the literature.^28^, ^34^ Such observation may be due to the synergistic effects of topographical cues and the sustained miR signaling using our fiber substrates. It should be noted, however, that for miR cocktails, there might also be antagonistic effects, which could affect neuronal outgrowth. This might explain why the four‐miR cocktail did not yield the best outcome.

To further evaluate the effects of these miRs/miR cocktails in promoting neurite outgrowth, the number of growth cones and growth cone area were quantified on 2D cultures. The choice of 2D cultures for the analyses of growth cone formation stems from the fact that as compared to aligned fiber substrates, the growth cone sprouts were completely extended on 2D cultures, hence facilitating visualization and quantification. So far, real‐time polymerase chain reaction (RT‐PCR) and growth cone evaluations were carried out to reveal the possible mechanisms of these miR cocktails in promoting neurite outgrowth. However, this work did not illustrate related pathways that may have been affected by these miR combinations and their possible antagonistic effects. Future works, such as immunostaining, western blot, and RNA sequencing, would be suitable to further elucidate these mechanisms in greater detail.

Based on our in vitro screening data, the top single, double, and triple miR cocktails were evaluated for their efficacy in vivo using SCI as a proof of principle. In doing so, we also analyzed if the biomimicry nature of the aligned fibers could provide good correlation between in vitro and in vivo outcomes. To enable the direct implantation of aligned fibers within the spinal cord, we utilized the fiber‐hydrogel system. This platform consisted of a collagen matrix that enabled the delivery of multiple biochemical factors (miRs, NT‐3).^58^ Most importantly, it also supported and retained aligned fibers in a 3D configuration to guide the direction of axon growth liken the in vitro situation. Consequently, we found that the trends of in vivo nerve regeneration closely resembled the in vitro results, particularly those involving CNS neurons. Specifically, miR‐132/miR‐222/miR‐431 generally provided the best regeneration outcomes as compared to miR‐21, miR‐222/miR‐431, and Neg miR treatments.

Comparing in vivo versus in vitro outcomes, we noted that the best in vitro candidate, miR‐222/miR‐431, revealed similar results as miR‐21, in terms of NF200 positive signal intensity inside the scaffolds after SCI (Figure 6A). On the other hand, the triple miRNA combination, miR‐132/miR‐222/miR‐431, showed significantly higher neurite density inside the scaffolds as compared to all the other groups (Figure 6A,B). One possible reason may be due to the enhanced growth cone formation and growth cone area in response to the triple miR cocktail as observed in Figure 4.

The potential of NT‐3 in promoting neuronal survival, axonal sprouting, and regeneration following SCI is well documented.^37^, ^38^, ^39^, ^40^ However, in our case, when NT‐3 was used alone to treat SCI, nerve ingrowth was observed but not robust (Figure 6A). In contrast, axonal growth was greatly improved when NT‐3 was coupled with miRs. These observations suggest that the mere modulation of the microenvironment by neurotrophins may be insufficient to achieve prominent nerve regeneration after nerve injuries. Conversely, by adding miRs to increase the intrinsic growth ability of axons, a significant regeneration was obtained (Figure 6B).

Through our systematic in vitro and in vivo studies, we have extended our knowledge on neuron intrinsic growth property and axon regeneration by using our aligned fiber screening platforms. Although SCI was used as a proof of principle to evaluate the in vivo efficacy of our aligned fiber construct, the promising outcomes observed at early time points of recovery suggests that this method holds tremendous potential for SCI treatment. Therefore, in‐depth studies such as tracing and functional evaluations will be conducted to further investigate the efficacy of our platform and the effects of these miRs on promoting functional recovery in the long term. Altogether, we have developed a robust nonviral gene delivery platform for both in vitro and in vivo studies, which addresses the problems of the lack of a delivery system and weak in vitro and in vivo correlations.

## 4 Conclusion

In this study, we established a biomimicking aligned fiber substrate that provides sustained and effective nonviral delivery of miRs for in vitro and in vivo miR screening. As compared to conventional 2D cultures, our aligned fiber substrates promoted longer neurite outgrowth and enhanced gene silencing in primary neurons. In addition, this platform could also be implantable for direct in vivo miR screening. As a proof of concept, complete transection SCI was carried out to evaluate the efficacy of this aligned fiber‐hydrogel system in vivo. Robust nerve ingrowth was observed as early as two weeks after scaffold implantation after SCI. Given the ability to deliver miRs nonvirally, our scaffolds may be translated to clinical applications without raising biosafety concerns associated with virus transfections. Studies are now ongoing to further evaluate the capability of our fiber‐hydrogel scaffolds in promoting nerve regeneration, remyelination, and functional recovery after SCI.

## 5 Experimental Section

*Materials*: Polycaprolactone (*M*w: 45 000 (45k PCL) and 80 000 (80k PCL)), DOPA, 2,2,2‐trifluoroethanol (TFE, ≥99.0%), poly‐d‐Lysine (PDL) (P0899), cytosine arabinoside (Ara‐C), 5‐fluoro‐2′‐deoxyuridine and Heparin sodium were purchased from Sigma‐Aldrich. Alexa‐Fluor 555 goat anti‐Mouse, Alexa‐Fluor 488 Phalloidin, Scrambled Neg miR, miR‐21‐5p (PM10206), miR‐132‐3p (PM10166), miR‐222‐3p (PM11376), and miR‐431‐5p (PM10091), laminin (23017015), DAPI (4′,6‐diamidino‐2‐phenylindole), paraformaldehyde (PFA, 7230681), phosphate buffered saline (PBS; pH7.4), SYBR Select Master Mix, Trizol Reagent, B‐27 supplement, N‐2 supplement, goat serum, neurobasal medium, Glutamax supplement, penicillin–streptomycin, bovine serum albumin (BSA, A1000801), and Quant‐iTTM RiboGreen RNA reagent kit (Invitrogen) were obtained from Life Technologies, USA. Cy5‐labelled double‐stranded RNA(Cy5‐RNA) of similar size as miR (i.e., 21–23 base pairs) and TKO were purchased from Integrated DNA Technologies (IDT) and MirusBio respectively. Mouse anti‐βIII Tubulin (Tuj‐1) (801202) and Chicken anti‐NF200 (822601) was purchased from Biolegend. Rabbit anti‐GFAP (Z0334) was obtained from DAKO. Rat‐tail Collagen type I was purchased from Corning. NT‐3 and nerve growth factor (NGF) were purchased from PeproTech. Dulbecco's modified Eagle medium/F12 (DMEM/F12) medium was purchased from Lonza, Switzerland. Fetal bovine serum (FBS) was acquired from Research Instruments.

*In Vitro Studies—Fabrication and Characterization of Aligned Fibers*: In vitro aligned fiber scaffolds were fabricated using the electrospinning process (Figure S6A, Supporting Information). Briefly, 50 mg of PCL (*M*_w_: 45 000, a.k.a. 45k PCL) was melted in an 18 mm × 18 mm mold at 60 °C before being cooled down to room temperature. The resulting block of 45k PCL polymer was then cut into pieces of 0.5 cm × 1.0 cm × 2.0 cm before they were sectioned into 20 µm thick films. These 45k PCL sheets were then washed three times in distilled water and placed in 1 × PBS at room temperature for immediate use.

45k PCL sheets were dried at 37 °C for 2 h prior to the electrospinning of fibers. These dried sheets were then sterilized with 70% ethanol before placing on glass coverslips (diameter: 18 mm) as shown in Figure S6B,C in the Supporting Information. Once placed in position, the coverslips were heated at 50 °C to designate a 10 mm × 10 mm cell seeding area before six of these coverslips were placed on a rotating wheel for the collection of electrospun fibers (Figure S6A, Supporting Information).

For electrospinning, PCL (*M*_w_: 80 000, a.k.a. 80k PCL) was dissolved in TFE to obtain a 14 wt% solution. The homogenous solution was then loaded into a syringe and dispensed at a fixed rate of 1.0 mL h^−1^ by a syringe pump (New Era pump Systems Inc., USA). Positive 8 kV (Gamma High Voltage, USA) and −4 kV were then applied to the polymer solution and the rotating collector (2400 rpm), respectively. The syringe and the collector were separated at 22 cm apart. Electrospun fibers were then collected on the coverslips and held in alignment by the melted 45k PCL sheets. Here, two different molecular weights of PCL were used due to their slight difference in melting point. Specifically, 45k *M*_w_ PCL sheets melted at 50 °C, which allowed them to be premelted onto the glass coverslips to serve as supports. At this temperature, the 80k *M*_w_ PCL fibers remained intact and were, hence, collected and stuck onto the 45k *M*_w_ PCL sheets.

The morphology of the aligned fiber substrates was evaluated by scanning electron microscopy (SEM) (JOEL, JSM‐6390LA, Japan) under an accelerating voltage of 10 kV after sputter coating with platinum for 100 s at 10 mA. The average fiber diameters were then quantified by measuring 100 fibers from high magnification images (2500 ×) using Image J software (National Institutes of Health (NIH), USA).

*In Vitro Studies—Preparation of miR‐Loaded PCL Fibers*: Suspended PCL fibers on coverslips were fitted into 12‐well plates and sterilized with 70% ethanol for 30 min. Thereafter, the fibers were immersed into 0.5 mg mL^−1^ DOPA that was dissolved in poly‐DOPA coating buffer (10 × 10^−3^ m bicine and 50 × 10^−3^ m NaCl, pH = 8.5) before placing on an orbital shaker (120 rpm) for 4 h. DOPA‐coated fibers were then washed with deionized water and lyophilized overnight. Subsequently, the lyophilized fibers were coated with 70 µg cm^−2^ of PDL for 1 h at 37 °C before rinsing off any unbound PDL with distilled water and coating these fibers with laminin at 7 µg cm^−2^ for 2 h at 37 °C.

1.5 µL of TransIT‐TKO was diluted in DMEM before complexation with 1 µg of miRs. The complexation was performed at room temperature for 10 min. After removing laminin, these complexes were placed on the 10 mm × 10 mm cell seeding area to allow complete adsorption at 37 °C for 2 h. All suspended PCL fibers were then divided into 18 groups, as shown in Table S1 in the Supporting Information.

*In Vitro Studies—In Vitro Characterization of miR‐Loaded Aligned Fibers*: To determine the distribution of miRs on the aligned fibers, Cy5‐RNA was complexed and coated using the same protocol as highlighted in Section 5.2.2. Two hours after coating, the scaffolds were rinsed once with PBS. The drug distribution was then observed with a fluorescent microscope (LeicaDMi8).

To evaluate the extent of cellular uptake, P1 cortical neurons were seeded onto Cy5‐RNA absorbed aligned fibers and cultured for 3 d. Following that, the colocalization of Cy5‐RNA, DAPI, and neuronal marker, βIII‐Tubulin, were examined under a confocal microscope (Zeiss LSM710).

To calculate microRNA loading efficiency, aligned fiber scaffolds (*n* = 3) with neg miR complex were washed once with 1 × PBS. The amount of unbound miR was then decomplexed by heparin (250 µg mL^−1^) before being determined by RiboGreen Assay. The fluorescence intensity was then measured by a microplate reader (Tecan, Infinite 200). MicroRNA loading efficiency was calculated based on the following equation

(1) $$\mathrm{Loading}\;\mathrm{efficiency}\left(\%\right)=\frac{\mathrm{Total}\;\mathrm{mass}\;\mathrm{of}\;\mathrm{miRs}-\mathrm{Mass}\;\mathrm{of}\;\mathrm{unbound}\;\mathrm{miRs}}{\mathrm{Total}\;\mathrm{mass}\;\mathrm{of}\;\mathrm{miRs}}\times 100\%$$

Thereafter, the miR‐loaded aligned fiber scaffolds were completely submerged in 1 mL of 1 × PBS and incubated at 37 °C. At each time point, 1 mL of supernatant was collected and an equal volume of fresh PBS was then added. The amount of miRs that were released at each time point were then determined using RiboGreen assay. Cumulative release profile was plotted as a percentage of the actual mass of miRs loaded on the scaffolds.

*In Vitro Studies—Primary Rat Neuron Isolation and Culture*: All animal experiments were approved by the Institutional Animal Care and Use Committee, Nanyang Technical University (IACUC, NTU).

*Cortical Neurons*: Dissociated cultures of rat cortical neurons were generated from either time‐mated embryonic day 14 (E14) rats or pups (P1). The cortices from either E14 or P1 were digested with 0.25% trypsin at 37 °C for 15 min. After homogenization, the suspension was passed through a 70 µm cell strainer (BD, Bioscience, USA) and the cells were seeded onto the miR‐loaded PCL scaffolds at a density of 25 000 cells scaffold^−1^. 2D PDL and laminin coated coverslips were set in parallel, onto which the neurons were transfection by 100 × 10^−9^ m miRs at 1:1 (v/v) of miR: TKO one day after cell seeding. All cultures were then maintained in Neurobasal media, which contained 10% FBS, 2% B‐27 Supplement, penicillin–streptomycin (10 µg mL^−1^), and 1% GlutaMAX. The medium was half changed 2 d after cell seeding and the culture process was maintained for 3 d in total. Neurons on scaffolds and coverslips were collected for immunofluorescent staining or real‐time PCR.

*Adult DRG Neurons*: Three adult Sprague‐Dawley rats were utilized and DRGs were collected in DMEM/F12 medium in a petri dish. All the meninges from the DRGs were taken off to minimize culture contamination with other cells. Dissociated cells were then seeded onto the miR‐loaded PCL scaffolds at a density of 5000 cells scaffold^−1^. The cultures were maintained at 37 °C and 5% CO_2_ in the DMEM/F12 medium containing 1:100 penicillin/streptomycin, 10% horse serum, 1% of N_2_ and 50 ng mL^−1^ of NGF. Half of the medium was changed 24 h after seeding and 10 µM per well of Ara‐C and 20 µM per well of 5‐fluoro‐2'‐deoxyuridine were added to the culture. Cultures were kept for 3 d postseeding.

*In Vitro Studies—Real‐Time PCR*: After 3 d of culture, P1 cortical neurons seeded on the scaffolds were lysed by TRIzol reagent and the RNA was extracted. The seeding density was 25 000 cells per scaffold and six scaffolds were pooled together. 500 ng of RNA was used for reverse transcription. Real‐time PCR was then carried out using SYBR Green Supermix in a StepOnePlus system (Applied Biosystems, USA). The sequences of the primers are shown in Table S2 in the Supporting Information and RNA18S was used as the housekeeping gene. All the primers showed similar amplification efficiency, hence the ΔΔ*C*_t_ method was used for fold change analysis. All results were normalized by the *C*_t_ value of neurons that were treated with Neg miR.

*In Vivo Studies—Fiber‐Hydrogel Scaffold Fabrication: Fibers*: Poly(caprolactone‐*co*‐ethyl ethylene phosphate) (PCLEEP) copolymer (*M*_w_ = 59 102, *M*_n_ = 25 542) was a gift from Dr. Yucai Wang's lab. It was synthesized as reported previously.^59^ PCLEEP was dissolved in TFE at 33% w/w and settled overnight before use to ensure homogeneity. A two‐pole air‐gap electrospinning technique was adopted to fabricate aligned PCLEEP fibers (Figure S6D, Supporting Information). Briefly, the electrospinning solution was loaded into a 3 mL syringe that was subsequently capped with a 21‐gauge blunt‐tipped needle. This needle tip was then charged with +8 kV and the two‐pole air‐gap collector was charged at −4 kV. The electrospinning solution was released at a flow rate of 1.5 mL h^−1^ by a syringe pump. PCLEEP fibers were then deposited within a 5.0 cm air gap area that was between the stationary poles. Each set of fibers was obtained after 6 min and 30 s of spinning and combined into set sets. Fibers were sterilized under UV light for 30 min before stacking the layers and rolling them into a bundle of fibers. A sterilized cylindrical mold (8.0 mm in length and 3.5 mm in inner diameter) was used to set the fiber bundles in the core region prior to the addition of collagen matrix.

*In Vivo Studies—Fiber‐Hydrogel Scaffold Fabrication: Collagen Matrix*: Rat‐tail type 1 collagen was used to fabricate the hydrogel matrix according to the manufacturer's protocol. Briefly, 10 × PBS, 1.0 N NaOH, deionized (DI) water, and collagen type 1 were added into a sterile 600 µL micro‐tube in the listed order and mixed gently to get a final collagen concentration of 3.0 mg mL^−1^. To promote infiltration of neurofilaments, growth factors were incorporated into this matrix. NT‐3 was reconstituted in 0.1% BSA and 400 µg µL^−1^ heparin at 1:1 v/v to arrive at a stock concentration of 2 µg µL^−1^. 4 µL of NT‐3 stock solution was then used to substitute 4 µL of DI water in the 250 µL collagen mixture per 8 mm mold. Hence, a total of 8 µg of NT‐3 was loaded into this mixture. 20 µg (7.5 µL of 100 × 10^−6^ m miR stock) of selected miRs were complexed with TKO (1:1 v/v) and subsequently loaded. A total of 2 µg of NT‐3 and 5 µg of miRs were used per animal. This collagen mixture was kept on ice until dispensed into the mold that contained the electrospun fiber bundle in the core region. Hydrogel formation took place at room temperature for 30 min before placing the scaffold at −20 °C for 4 h prior to overnight lyophilization. Scaffolds were cut into 2 mm long under sterilized conditions before implantation into each animal.

*In Vivo Studies—Spinal Cord Transection and Scaffold Implantation*: Female Sprague‐Dawley rats (7–9 weeks, 200–250 g) were obtained from In Vivos Pte Ltd (Singapore). Rats were anesthetized with an intraperitoneal injection of ketamine (73 mg kg^−1^) and xylazine (7.3 mg kg^−1^). All animals were injected with buprenorphine subcutaneously (0.05 mg kg^−1^) before the surgery. The surgical field was shaved and cleaned with 70% ethanol and treated with betadine. The skin was incised above the thoracic level, and the muscles were moved apart to expose the vertebra at level T8–T11. A dorsal laminectomy was performed on T9–T10. Dura was cut open and 2 mm of the spinal cord was removed using fine micro scissors. A 2.0 mm long fiber‐hydrogel scaffold was then implanted to reconnect the rostral and caudal parts of the resected spinal cord. Afterward, the dura was sutured, and a 50 µm thick PCL film was put above the spinal cord to cover the injury area. The muscles were then sutured and the skin was closed with wound clips. Animals were randomly divided into five treatment groups as presented in Table S3 in the Supporting Information.

*In Vivo Studies—Immunohistochemistry: In Vitro*: After culturing for 3 d, neurons were fixed with 4% PFA for 30 min. After washing in 0.1 m PBS for three times (5 min each), the cells were permeabilized in 0.1% Triton X‐100 in 0.1 m PBS for 15 min. Thereafter, the samples were incubated in nonspecific blocking solution (5% goat serum) for 1 h at room temperature, followed by incubation with primary antibody, mouse‐anti βIII‐Tubulin (Tuj‐1, 1:1000), overnight at 4 °C. Following that, the cells were washed and detected with Alexa Fluor 546 fluorescent secondary antibodies (1:1000) at room temperature for 2 h. The nuclei were counterstained with DAPI. Cortical neurons cultured on 2D glass coverslips were stained with Tuj‐1, Phallodin‐488 (1:500), and DAPI.

For cortical neurons cultured on the scaffolds, 80 cells were quantified in each trial and 240 neurons were counted in total. For adult DRG neurons cultured on the scaffolds, 50 solitary cells from each group were imaged and quantified. Neurons were counted in terms of total length and the longest neurite length in each group. Three biological repeats were carried out for each experimental group of both cortical neuron and DRG neuron cultures.

For cortical neurons cultured on 2D, 40 cells imaged from three glass coverslips for each experimental group were quantified in terms of growth cone numbers and the growth cone area per neuron. All the quantifications were done using the ImageJ software.

*In Vivo Studies—Immunohistochemistry: In Vivo*: At 14 d postinjury, animals were perfused with 0.9% saline followed by 4% ice‐cold PFA. After perfusion, 1.5 cm of spinal cords containing the injury site were dissected and postfixed for 2 h before transferring to 15% sucrose for 24 h followed by 30% sucrose at 4 °C until cryosectioned. Spinal cord samples were sectioned into 20 µm thick horizontal sections and directly mounted on glass slides. The frozen sections were blocked with 10% goat serum and incubated for at least 1 h in a humidified box. The following primary antibodies were used: chicken anti‐NF200 (1:1000) and rabbit anti‐GFAP (1:1000). Samples were subsequently washed three times with PBS and incubated with the following secondary antibodies: Alexa Fluor 555‐conjugated Goat Anti‐Chicken (1:1000) and Alexa Fluor 488‐conjugated Goat Anti‐Rabbit (1:700) for 1.5 h. Nuclear staining was performed by incubating the sections with DAPI (1:1000) at room temperature for 10 min after the secondary antibodies. All samples were finally examined using a fluorescent inverted microscope (Leica DMi8).

For tissue samples, stitched images of the injury site were taken under 10x magnification. For nerve ingrowth measurements, the percentage of neurofilament area occupied in the entire scaffold was quantified.

For glial scar measurement, the percent area of GFAP^+^ signal within 250 µm from interface of the injury site was quantified. The GFAP^+^ signals were correlated to pixel intensity of fluorescent images. The images were converted to eight‐bit and thresholded to segregate GFAP^+^ signals and background. All images were taken under the same setting. All quantifications were done using the ImageJ software.

*Statistical Analyses*: One‐way analysis of variance (ANOVA) and Tukey post hoc test was used when the data were normally distributed and had equal variances. For data that were not normally distributed or had unequal variances, Kruskal–Wallis and Mann–Whitney *U*‐test was used for comparison between more than two groups. For comparison between two groups, Student's *t*‐test was used. All values, unless mentioned otherwise, were represented as mean ± S.E.M.

## Conflict of Interest

The authors declare no conflict of interest.

## Supporting information

Supplementary
