# Biomimicking Fiber Scaffold as an Effective In Vitro and In Vivo MicroRNA Screening Platform for Directing Tissue Regeneration

**DOI:** 10.1002/advs.202201954

**Published:** 2022-05-16

**Authors:** Na Zhang, Ulla Milbreta, Jiah Shin Chin, Coline Pinese, Junquan Lin, Hitomi Shirahama, Wei Jiang, Hang Liu, Ruifa Mi, Ahmet Hoke, Wutian Wu, Sing Yian Chew

*Adv. Sci*. **2019**, *6*, 1800808

DOI: 10.1002/advs.201800808

In the original published article, in the Methods subsection ‘*In Vivo Studies—Fiber‐Hydrogel Scaffold Fabrication*’ 15 µl of 100 × 10^−3^ m of miR stock, instead of 7.5 µl, was added to achieve a total mass of 20 µg miR loaded into the collagen mixture. This typo error was spotted after revisiting the calculations. We confirmed that the absolute mass of miR received by each animal and the absolute mass of miR added into the collagen mixture, as stated in the original paper, are both correct. The authors apologize for any inconvenience this may have caused.

