# Supplementary material for: Biomimicking Fiber Scaffold as an Effective In Vitro and In Vivo MicroRNA Screening Platform for Directing Tissue Regeneration
Source: Adv Sci (Weinh). 2019 Feb 27;6(9):1800808. doi: 10.1002/advs.201800808 (PMC6498117; doi:10.1002/advs.201800808)
Supplement: Supplementary file 1 — Supplementary [file ADVS-6-1800808-s001.pdf]

Copyright WILEY-VCH Verlag GmbH & Co. KGaA, 69469 Weinheim, Germany, 2018.

ADVANCED SCIENCE
Open Access

Supporting Information

for *Adv. Sci.,* DOI: 10.1002/advs.201800808

Biomimicking Fiber Scaffold as an Effective In Vitro and In Vivo MicroRNA Screening Platform for Directing Tissue Regeneration

*Na Zhang, Ulla Milbreta, Jiah Shin Chin, Coline Pinese, Junquan Lin, Hitomi Shirahama, Wei Jiang, Hang Liu, Ruifa Mi, Ahmet Hoke, Wutian Wu, and Sing Yian Chew**

Table 1. MicroRNA cocktails and denotations

	Groups	Denotation
Controls	No TKO-miR complexes	Plain
	TKO with scrambled negative miR	Neg miR
	TKO only	TKO
Single miR	TKO with miR-21	miR-21
	TKO with miR-132	miR-132
	TKO with miR-222	miR-222
	TKO with miR-431	miR-431
2-miR cocktails	TKO with equal masses of miR-21 and miR-132	miR-21/miR-132
	TKO with equal masses of miR-21 and miR-222	miR-21/miR-222
	TKO with equal masses of miR-21 and miR-431	miR-21/miR-431
	TKO with equal masses of miR-132 and miR-222	miR-132/miR-222
	TKO with equal masses of miR-132 and miR-431	miR-132/miR-431
	TKO with equal masses of miR-222 and miR-431	miR-222/miR-431
3-miR cocktails	TKO with equal masses of miR-21, miR-132 and miR-222	miR-21/miR-132/miR-431
	TKO with equal masses of miR-21, miR-132 and miR-431	miR-21/miR-132/miR-222
	TKO with equal masses of miR-21, miR-222 and miR-431	miR-21/miR-222/miR-431
	TKO with equal masses of miR-132, miR-222 and miR-431	miR-132/miR-222/miR-431
4-miR cocktails	TKO with equal masses of miR-21, miR-132, miR-222 and miR-431	4-miRs

Table 2. Primer sequences used for real-time PCR

18S	Forward	5'-GCAATTATTCCCCATGAACG-3'
	Reverse	5'-GGCCTCACTAAACCATCCAA-3'
Sprouty2	Forward	5'-TGGCAAGTGCAAGTGTAAGG-3'
	Reverse	5'-ACCATCGCGTACAACAGTGA-3'
PTEN	Forward	5'-CACAAGAGGCCCTGGATTTTTATG-3'
	Reverse	5'-CATAGCGCCTCTGACTGGGAATAG-3'
Rasa1	Forward	5'-CAGGATCTCTGCGTGTTCGA-3'
	Reverse	5'-TGTTCGGTCTTGTCCACATACG-3'
Kremen1	Forward	5'-CAGAGTGGAAGGATGGACCG-3'
	Reverse	5'-CGTCACGTGCAGAAGGATCT-3'

Table 3. Description of *in vivo* treatment and sample sizes

Group	Description	Sample size
NT-3	Animals received NT-3 encapsulated scaffolds	3
Neg-miR + NT-3	Animals received Neg-miR and NT-3 encapsulated scaffolds	4
miR-21 + NT-3	Animals received miR-21 and NT-3 encapsulated scaffolds	3
miR-222/miR-431 + NT-3	Animals received miR-222/-431 and NT-3 encapsulated scaffolds	4
miR-132/miR-222/miR-431 + NT-3	Animals received miR-132/-222/-431 and NT-3 encapsulated scaffolds	3

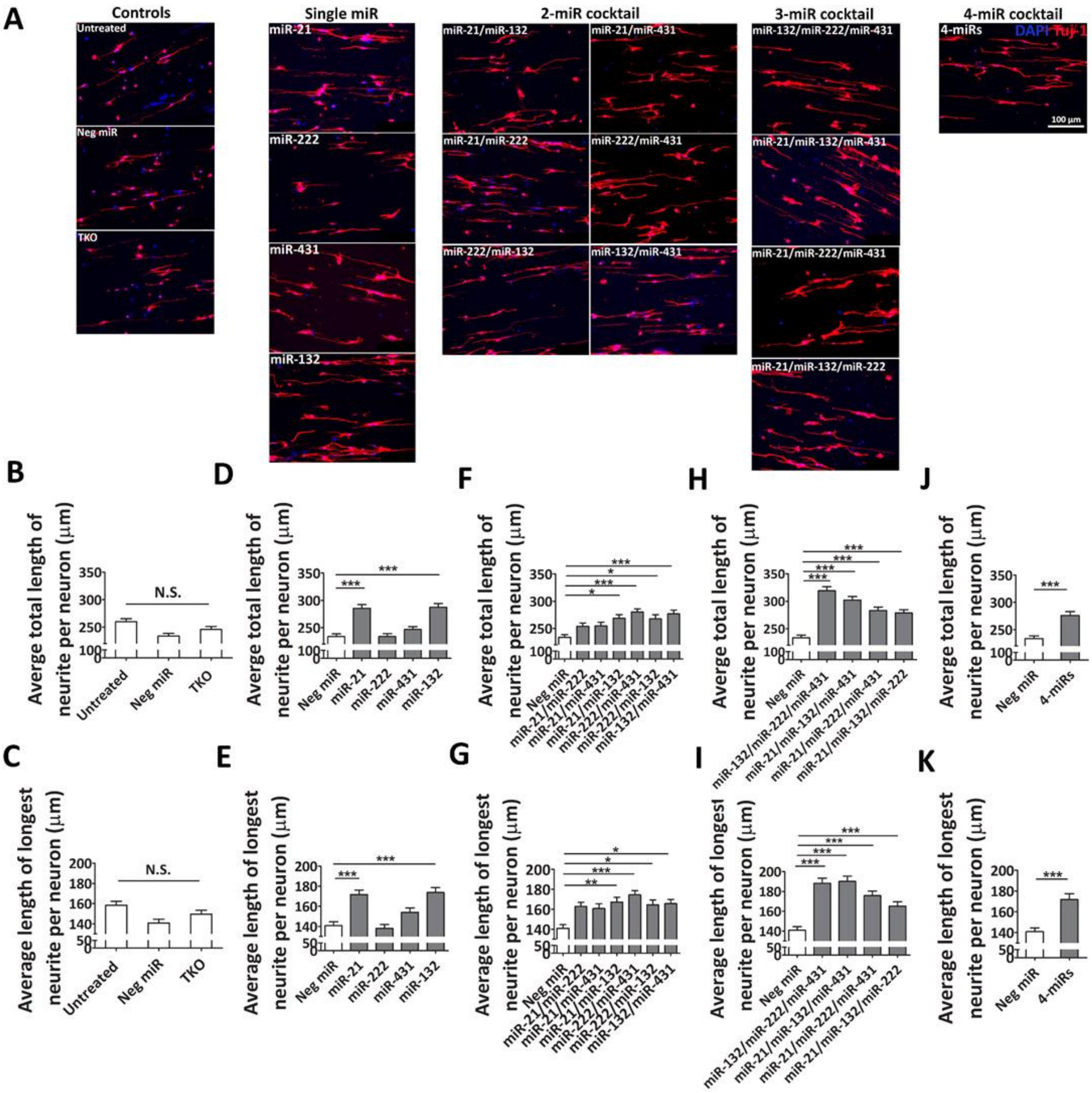

Supplementary Figure 1. ***In vitro*** **miR cocktail screening using E14 cortical neurons.** (A)Representative fluorescent microscopy images of untreated cells and cells treated with Neg miR, TKO, single miR, 2-miR cocktails, 3-miR cocktails and 4-miR cocktail. (B-K) Average total neurite length and average length of the longest neurite of each group. Data shown as mean ± S.E.M. *p < 0.05, **p < 0.01, ***p < 0.001, one-way ANOVA.

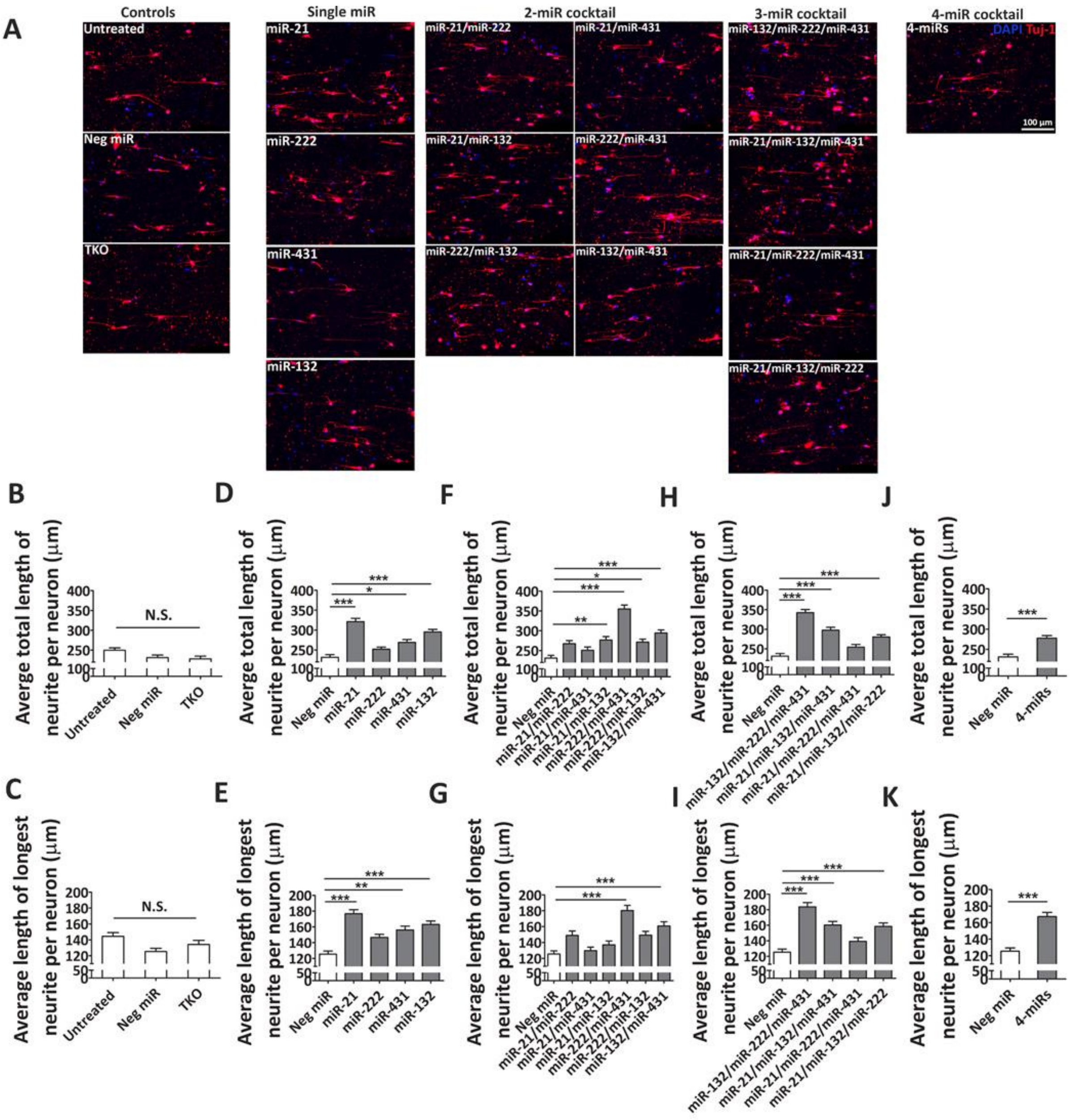

Supplementary Figure 2. ***In vitro*** **miR cocktail screening using P1 cortical neurons.** (A) Representative fluorescent microscopy images of untreated cells and cells treated with Neg miR, TKO, single miR, 2-miR cocktails, 3-miR cocktails and 4-miR cocktail. (B-K) Average total length of neurite and average length of the longest neurite of each group. Data presented as mean ± S.E.M, *p < 0.05, **p < 0.01, ***p < 0.001, one-way ANOVA.

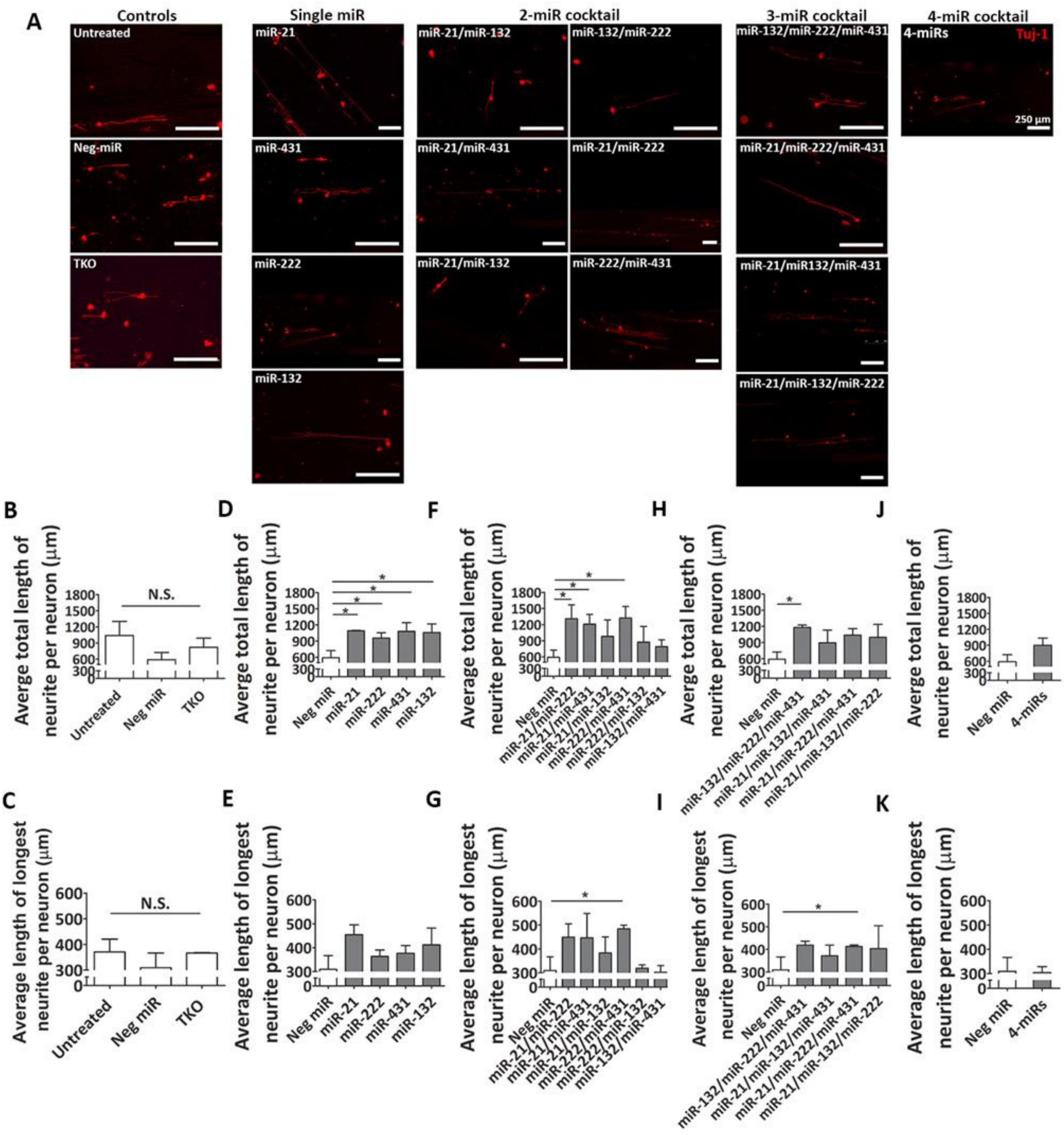

Supplementary Figure 3. *In vitro* miR cocktail screening using adult DRG neurons. (A) Representative fluorescent microscopy images of untreated cells and cells treated with Neg miR, TKO, single miR, 2-miR cocktails, 3-miR cocktails and 4-miR cocktail. (B-K) Average total length of neurite *and* average length of the longest neurite of each group. Data shown as mean ± S.E.M, *p < 0.05, Shapiro-Wilk normality test followed by Kruskal-Wallis test and Mann-Whitney post hoc test.

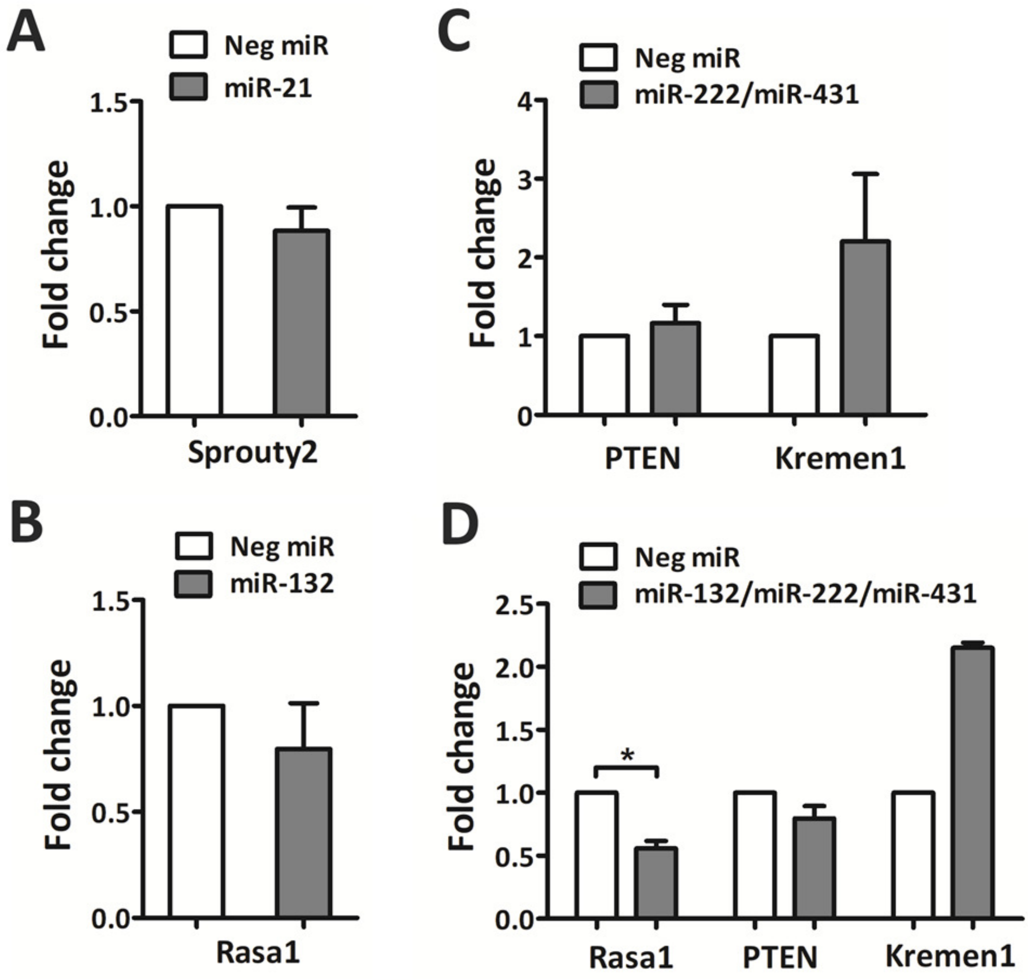

Supplementary Figure 4. *In vitro* gene silencing at Day 3 after bolus delivery of miRs to 2D cultures of P1 cortical neurons. (A-D) Fold changes after miR treatment show that bolus transfection of individual miR and miR cocktails did not induce significant gene silencing. All comparisons were normalized with Neg miR-treated group. Data presented as mean ± S.E.M, $*p < 0.05$, Student t-test.

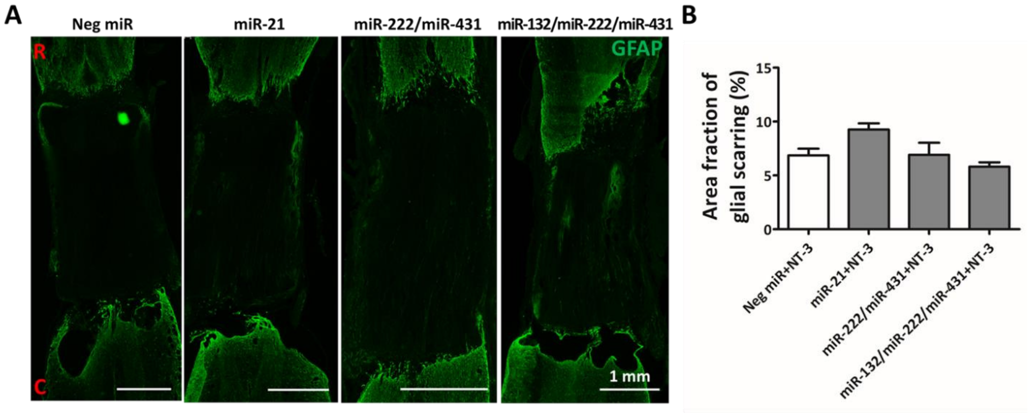

Supplementary Figure 5. No difference in *in vivo* glial scarring after SCI and treatment with different miR combinations (A) Representative fluorescent microscopy images of GFAP (green) expression at injury sites in Neg miR, miR-21, miR-222/miR-431 and miR-132/miR-222/miR-431 groups. (B) Percent area occupied by GFAP positive signals, showing no significant difference in glial scar formation after treatment with different miR cocktails. Data represented as mean ± S.E.M., Shapiro-Wilk normality test followed by Kruskal-Wallis test and Mann-Whitney post hoc test.

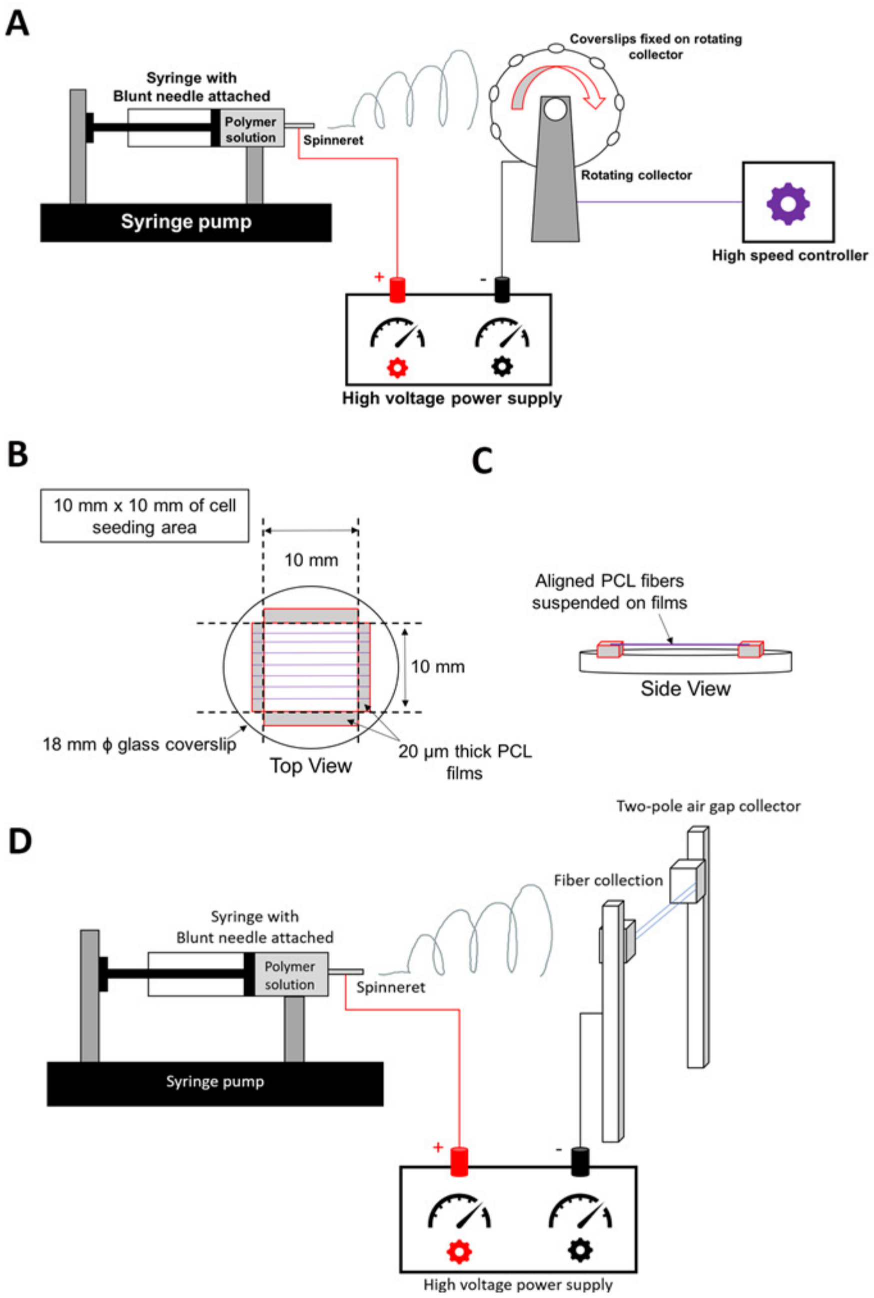

Supplementary Figure 6. Fabrication of aligned fiber scaffolds. (A) Schematic diagram of electrospinning with a rotating collector for preparation of *in vitro* scaffolds. (B) The top view of 18 mm diameter glass coverslips where four 45k PCL sheets were placed in position to designate a 10 mm × 10 mm cell seeding area. (C) Side view to show placement and suspension of electrospun fibers by 45k PCL sheets. (D) Schematic diagram of electrospinning with a two-pole air gap collector for preparation of *in vivo* scaffolds.
